# Non-contiguous finished genome sequence and description of *Clostridium saudii* sp. nov

**DOI:** 10.1186/1944-3277-9-8

**Published:** 2014-12-08

**Authors:** Emmanouil Angelakis, Fehmida Bibi, Dhamodharan Ramasamy, Esam I Azhar, Asif A Jiman-Fatani, Sally M Aboushoushah, Jean-Christophe Lagier, Catherine Robert, Aurelia Caputo, Muhammad Yasir, Pierre-Edouard Fournier, Didier Raoult

**Affiliations:** 1 Unité de Recherche sur les Maladies Infectieuses et Tropicales Emergentes, UMR CNRS, Institut Hospitalo-Universitaire Méditerranée-Infection, Faculté de médecine, Aix-Marseille Université, Marseille, France; 2 Special Infectious Agents Unit, King Fahd Medical Research Center, King Abdulaziz University, Jeddah, Saudi Arabia; 3 Department of Medical Laboratory Technology, Faculty of Applied Medical Sciences, King Abdulaziz University, Jeddah, Saudi Arabia; 4 Department of Medical Microbiology and Parasitology, Faculty of Medicine, King Abdulaziz University, Jeddah, Saudi Arabia

**Keywords:** *Clostridium saudii*, Genome, Culturomics, Taxono-genomics

## Abstract

*Clostridium saudii* strain JCC^T^ sp. nov. is the type strain of *C. saudii* sp. nov., a new species within the genus *Clostridia*. This strain, whose genome is described here, was isolated from a fecal sample collected from an obese 24-year-old (body mass index 52 kg/m2) man living in Jeddah, Saudi Arabia. *C. saudii* is a Gram-positive, anaerobic bacillus. Here we describe the features of this organism, together with the complete genome sequence and annotation. The 3,653,762 bp long genome contains 3,452 protein-coding and 53 RNA genes, including 4 rRNA genes.

## Introduction

*Clostridium saudii* strain JCC^T^ (=CSUR P697 = DSM 27835) is the type strain of *C. saudii* sp. nov. This bacterium is a Gram-positive, anaerobic, spore-forming indole negative bacillus that was isolated from the stool sample of an obese 24 year-old Saudi individual, as a part of a culturomics study as previously reported [1-3].

The current prokaryote species classification method, known as polyphasic taxonomy, is based on a combination of genomic and phenotypic properties [4]. The usual parameters used to delineate a bacterial species include 16S rDNA sequence identity and phylogeny [2,3], genomic G + C content diversity and DNA–DNA hybridization (DDH) [4,5]. Nevertheless, some limitations appear, notably because the cutoff values vary dramatically between species and genera [6]. The introduction of high-throughput sequencing techniques has made genomic data for many bacterial species available [7]. To date, more than 4,000 bacterial genomes have been published and approximately 15,000 genomes project are anticipated to be completed in a near future [5]. We recently proposed a new method (taxono-genomics), which integrates genomic information in the taxonomic framework, combining phenotypic characteristics, including MALDI-TOF MS spectra, and genomic analysis [8-38].

The genus *Clostridium* was first created in 1880 [39] and consists of obligate anaerobic rod-shaped bacilli able to produce endospores [39]. To date, more than 200 species have been described (http://www.bacterio.cict.fr/c/clostridium.html). Members of the genus *Clostridium* are mostly environmental bacteria or associated with the commensal digestive flora of mammals. However, *C. botulinum*, *C. difficile* and *C. tetani* are major human pathogens [39].

## Classification and features

**Figure 1:**
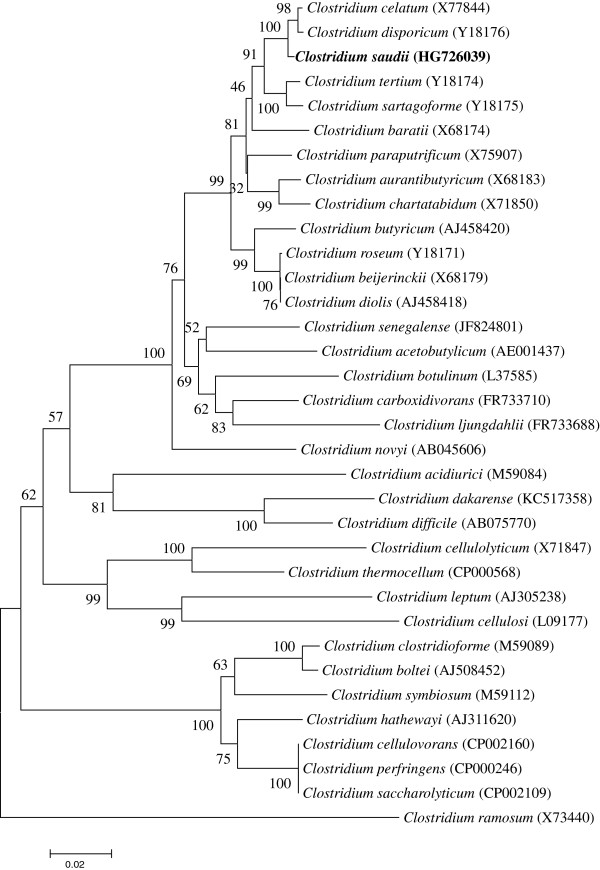
**A consensus phylogenetic tree highlighting the position of *Clostridium saudii *strain JCC**^**T **^**relative to other type strains within the genus *Clostridium*.** GenBank accession numbers are indicated in parentheses. Sequences were aligned using CLUSTALW, and phylogenetic inferences obtained using the maximum-likelihood method in the MEGA software package. Numbers at the nodes are percentages of bootstrap values from 500 replicates that support the node. *Clostridium ramosum* was used as the outgroup. The scale bar represents 2% nucleotide sequence divergence.

A stool sample was collected from an obese 24-year-old male Saudi volunteer patient from Jeddah. The patient gave an informed and signed consent, and the agreement of the local Ethical Committee of the King Abdulaziz University, King Fahd medical Research Centre, Saudi Arabia, and of the local ethics committee of the IFR48 (Marseille, France) were obtained under agreement number 014-CEGMR-2-ETH-P and 09–022 respectively. The fecal specimen was preserved at −80°C after collection and sent to Marseille. *C. saudii* strain JCC^T^ (Table 1) was isolated in July 2013 by anaerobic cultivation on 5% sheep blood-enriched Columbia agar (BioMerieux, Marcy l’Etoile, France) after a 5-day preincubation on blood culture bottle with rumen fluid. This strain exhibited a 98.3% nucleotide sequence similarity with *Clostridiumdisporicum* (Y18176) (Figure 1). This value was lower than the 98.7% 16S rRNA gene sequence threshold recommended by Stackebrandt and Ebers to delineate a new species without carrying out DNA-DNA hybridization [3] and was in the 78.4 to 98.9% range of 16S rRNA identity values observed among 41 *Clostridium* species with validly published names [40].

**Table 1:** **Classification and general features of *Clostridium saudii *** **strain JCC**^**T**^

**MIGS ID**	**Property**	**Term**	**Evidence code**^**a**^
	Current classification	Domain *Bacteria*	TAS [41]
		Phylum *Firmicutes*	TAS [42-44]
		Class *Clostridia*	TAS [45,46]
		Order *Clostridiales*	TAS [47,48]
		Family *Clostridiaceae*	TAS [47,49]
		Genus *Clostridium*	IDA [47,50,51]
		Species *Clostridium saudii*	IDA
		Type strain JCC^T^	IDA
	Gram stain	Positive	IDA
	Cell shape	Rod	IDA
	Motility	Motile	IDA
	Sporulation	Sporulating	IDA
	Temperature range	Mesophile	IDA
	Optimum temperature	37°C	IDA
MIGS-6.3	Salinity	Unknown	IDA
MIGS-22	Oxygen requirement	Anaerobic	IDA
	Carbon source	Unknown	IDA
	Energy source	Unknown	IDA
MIGS-6	Habitat	Human gut	IDA
MIGS-15	Biotic relationship	Free living	IDA
	Pathogenicity	Unknown	
	Biosafety level	2	
MIGS-14	Isolation	Human feces	
MIGS-4	Geographic location	Jeddah, Saudi Arabia	IDA
MIGS-5	Sample collection time	July 2013	IDA
MIGS-4.1	Latitude	21.422487	IDA
MIGS-4.1	Longitude	39.856184	IDA
MIGS-4.3	Depth	surface	IDA
MIGS-4.4	Altitude	0 m above sea level	IDA

Evidence codes - IDA: Inferred from Direct Assay; TAS: Traceable Author Statement (i.e., a direct report exists in the literature); NAS: Non-traceable Author Statement (i.e., not directly observed for the living, isolated sample, but based on a generally accepted property for the species, or anecdotal evidence). These evidence codes are from of the Gene Ontology project [52]. If the evidence is IDA, then the property was directly observed for a live isolate by one of the authors or an expert mentioned in the acknowledgements.

**Figure 2:**
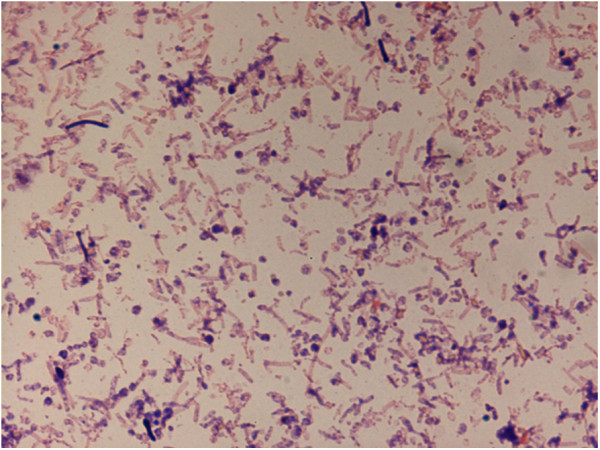
**Gram stain of *Clostridium saudii *** **strain JCC**^**T**^**.**

**Figure 3:**
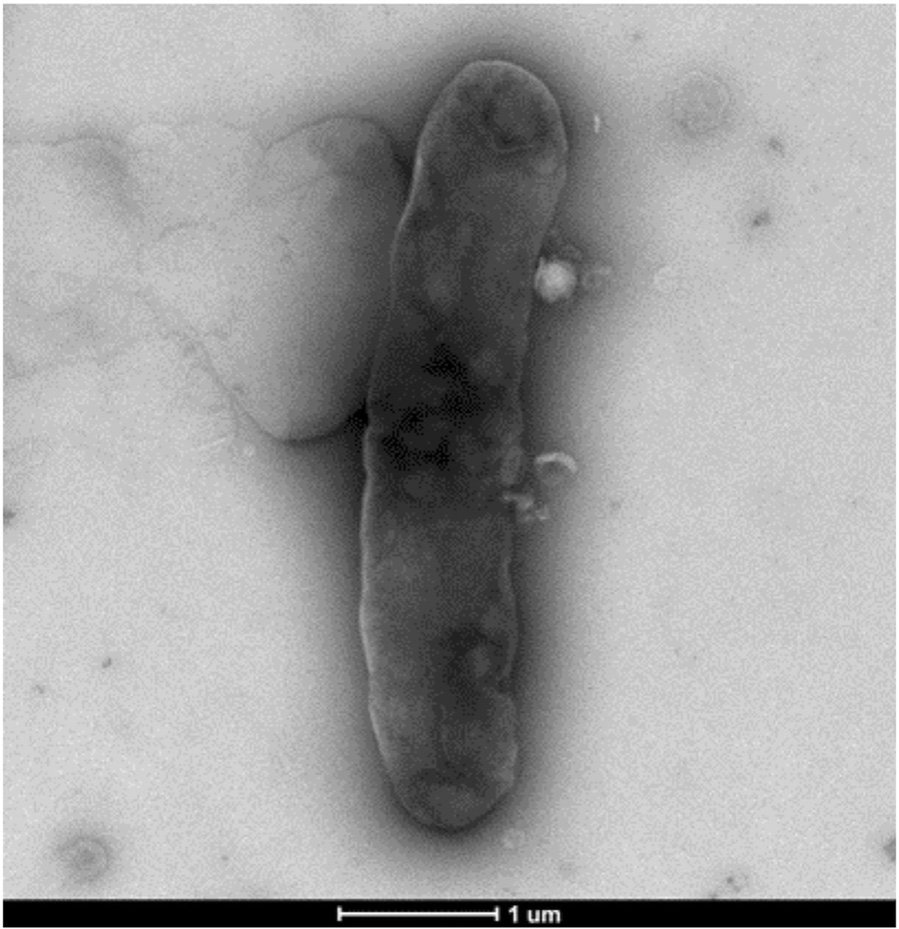
**Transmission electron micrograph of *C. saudii *strain JCC**^**T**^**, taken using a Morgani 268D (Philips) at an operating voltage of 60 kV. **The scale bar represents 1 um.

For the growth of *C. saudii* we tested four temperatures (25, 30, 37, 45°C); growth occurred between 25 and 37°C, however optimal growth occurred at 37°C, 24 hours after inoculation. No growth occurred at 45°C. Colonies were translucent on 5% sheep blood-enriched Columbia agar (BioMerieux). Colonies on blood-enriched Columbia agar were about 0.2 to 0.3 mm in diameter and translucent light grey. Growth of the strain was tested in the same agar under anaerobic and microaerophilic conditions using GENbag anaer and GENbag microaer systems, respectively (BioMerieux), and in aerobic conditions, with or without 5% CO_2_. Growth was observed only under anaerobic conditions and no growth occurred under aerobic or microaerophilic conditions. Gram staining showed Gram-positive rods able to form spores (Figure 1) and the motility test was positive. Cells grown on agar exhibit a mean diameter of 1 μm and a mean length of 1.22 μm in electron microscopy (Figure 2, Figure 3).

*C. saudii* did not have catalase or oxidase activity (Table 2). On an API Rapid ID 32A strip (BioMerieux), *C. saudii* presented positive reactions for α-galactosidase, β-galactosidase, β-galactosidase-6-phosphatase, α-glucosidase, β-glucosidase, α-arabinosidase, N-acetyl-β-glucosaminidase, alkaline phosphatase, arginine arylamidase, pyroglutamic acid arylamidase, tyrosine arylamidase, alanine arylamidase, glycine arylamidase and histidine arylamidase. Negative reactions were obtained for urease, arginine dihydrolase, β-glucuronidase, fermentation of mannose and raffinose, glutamic acid decarboxylase, α-fucosidase, nitrate reduction, indole production, proline arylamidase, leucyl glycine arylamidase, phenylalanine arylamidase, leucine arylamidase, glutamyl glutamic acid arylamidase and serine arylamidase. *C. saudii* was asaccharolytic on an API 50CH strip (Biomerieux). *C. saudii* is susceptible imipenem, trimethoprim-sulfamethoxazole, metronidazole, doxycycline, rifampicin, vancomycin and amoxicillin-clavulanate and resistant to amoxicillin, ciprofloxacine, erythromycin and gentamicin. The differential phenotypic characteristics with other *Clostridium* species are summarized in Table 2.

**Table 2:** **Differential characteristics of *Clostridium saudii *** **JCC**^**T**^**, *C. beijerinckii* strain NCIMB 8052, *C. disporicum *** **NCIB 12424, *C. carboxidivorans *** **strain P7, *C. senegalense* strain JC122, *C. dakarense *** **strain FF1 and *C. difficile* strain B1**

**Properties**	***C. saudii***	***C. beijerinckii***	***C. disporicum***	***C. carboxidivorans***	***C. senegalense***	***C. dakarense***	***C. difficile***
Cell diameter (μm)	1.0	1.7	1.5	1.5	1.1	1.2	3.0
Oxygen requirement	Strictly	Strictly	Strictly	Strictly	Strictly	Strictly	Strictly
	anaerobic	anaerobic	anaerobic	anaerobic	anaerobic	anaerobic	anaerobic
Gram stain	Positive	Variable	Positive	Positive	Positive	Positive	Variable
Motility	Motile	Motile	Na	Motile	Motile	Motile	Motile
Endospore formation	+	+	Na	+	+	+	+
Indole	-	Na	-	-	-	+	Na
**Production of**							
Alkaline phosphatase	-	Na	Na	Na	-	+	Na
Catalase	-	-	-	-	-	-	Na
Oxidase	-	Na	Na	-	-	-	Na
Nitrate reductase	-	-	Na	-	-	-	-
Urease	-	-	Na	-	-	-	Na
β-galactosidase	-	Na	Na	Na	-	-	Na
N-acetyl-glucosamine	-	Na	Na	Na	+		Na
**Acid from**							
L-Arabinose	-	+	Na	+	Na	-	-
Ribose	-	-	+	+	Na	-	-
Mannose	-	+	+	+	Na	-	+
Mannitol	-	+	+	+	Na	-	+
Sucrose	-	+	+	+	Na	-	+
D-glucose	-	+	+	+	Na	+	Na
D-fructose	-	+	+	+	Na	-	+
D-maltose	-	+	+	+	Na	+	-
D-lactose	-	+	+	+	Na	-	-
**G + C content (%)**	28	28	29	31	26.8	27.98	28
**Habitat**	Human gut	Human gut	Rat gut	Environment	Human gut	Human gut	Human gut

*na* = data not available; *w* = weak.

**Figure 4:**
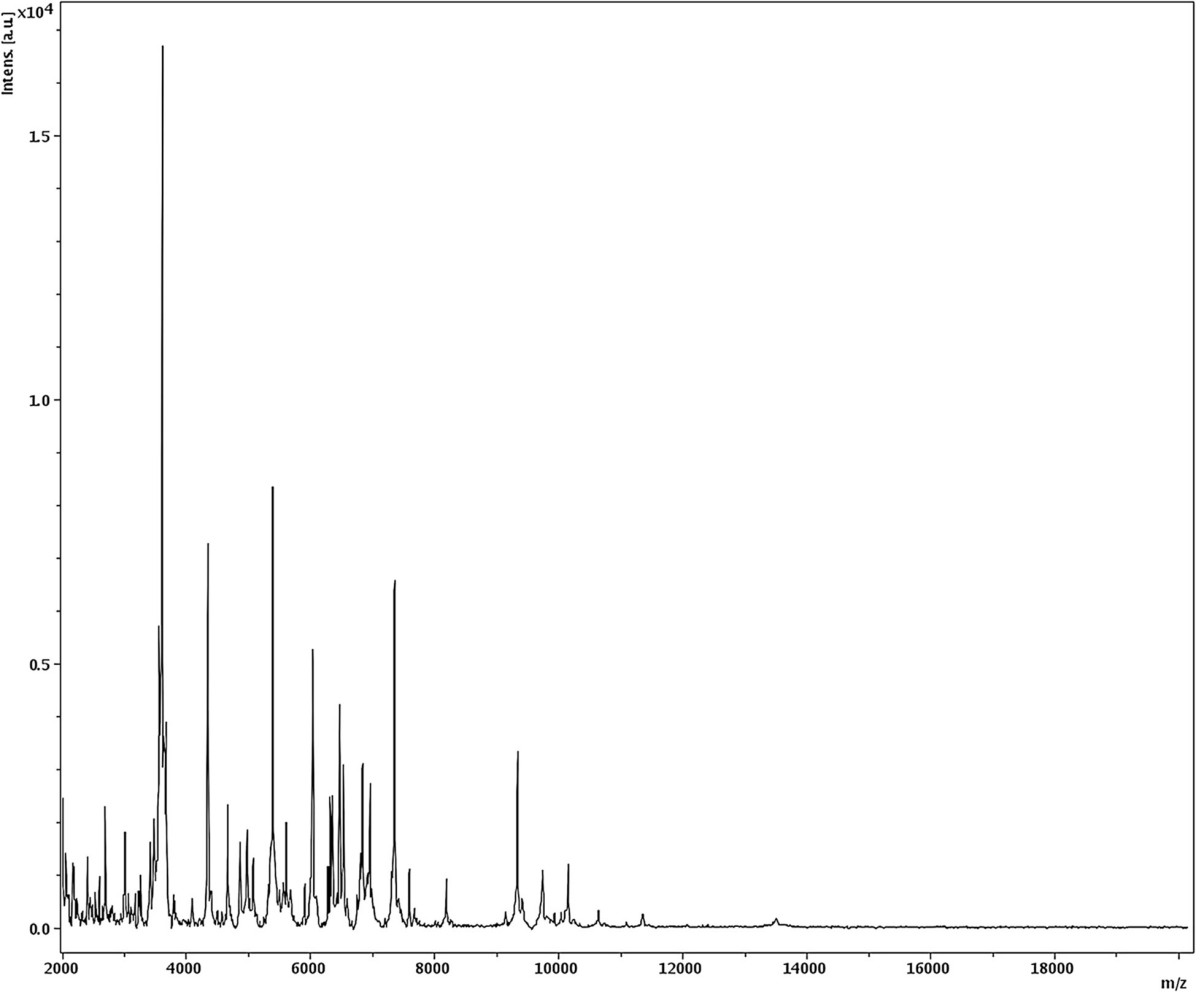
**Reference mass spectrum from *C. saudii *strain JCC**^**T**^**.** Spectra from 12 individual colonies were compared and a reference spectrum was generated.

**Figure 5:**
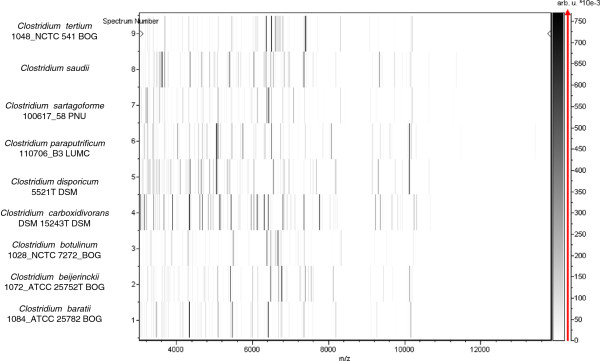
**Gel view comparing spectra from *Clostridium saudii *strain JCC**^**T**^**, *Clostridium tertium*, *Clostridium sartagoforme*, *Clostridium baratii*, *Clostridium beijerinckii*, *Clostridium botulinum*, *Clostridium carboxidivorans *and *Clostridium paraputrificum*.** The gel view presents the raw spectra of loaded spectrum files as a pseudo-electrophoretic gel. The x-axis records the m/z value. The left y-axis displays the running spectrum number originating from subsequent spectra loading. The peak intensity is expressed by a grey scale scheme code. The grey scale bar on the right y-axis indicates the relation between the shade of grey a peak is displayed with and the peak intensity in arbitrary units. Species are listed on the left.

Matrix-assisted laser-desorption/ionization time-of-flight (MALDI-TOF) MS protein analysis was carried out as previously described [53]. Briefly, a pipette tip was used to pick one isolated bacterial colony from a culture agar plate and spread it as a thin film on a MTP 384 MALDI-TOF target plate (Bruker Daltonics, Leipzig, Germany). Twelve distinct deposits from twelve isolated colonies were performed for *C. saudii* JCC^T^. Each smear was overlaid with 2 μL of matrix solution (saturated solution of alpha-cyano-4-hydroxycinnamic acid) in 50% acetonitrile, 2.5% tri-fluoracetic acid, and allowed to dry for 5 minutes. Measurements were performed with a Microflex spectrometer (Bruker). Spectra were recorded in the positive linear mode for the mass range of 2,000 to 20,000 Da (parameter settings: ion source 1 (ISI), 20 kV; IS2, 18.5 kV; lens, 7 kV). A spectrum was obtained after 675 shots with variable laser power. The time of acquisition was between 30 seconds and 1 minute per spot. The twelve JCC^T^ spectra were imported into the MALDI BioTyper software (version 2.0, Bruker) and analyzed by standard pattern matching (with default parameter settings) against the main spectra of 3,769 bacteria, including 228 spectra from 96 *Clostridium* species. The method of identification included the m/z from 3,000 to 15,000 Da. For every spectrum, a maximum of 100 peaks were compared with spectra in database. The resulting score enabled the identification of tested species, or not: a score ≥ 2 with a validly published species enabled identification at the species level, a score ≥ 1.7 but < 2 enabled identification at the genus level, and a score < 1.7 did not enable any identification. No significant MALDI-TOF score was obtained for strain JCC^T^ against the Bruker database, suggesting that our isolate was not a member of a known species. We added the spectrum from strain JCC^T^ to our database (Figure 4). Finally, the gel view showed the spectral differences with other members of the genus *Clostridium* (Figure 5).

## Genome sequencing information

### Genome project history

The organism was selected for sequencing on the basis of its phylogenetic position and 16S rRNA similarity to members of the genus *Clostridium*, and is part of a study of the human digestive flora aiming at isolating all bacterial species in human feces [1]. It was the 101^st^ genome of a *Clostridium* species and the first genome of *C. saudii* sp. nov. The GenBank accession number is HG726039 and consists of 104 contigs. Table 2 shows the project information and its association with MIGS version 2.0 compliance [54].

### Growth conditions and DNA isolation

*C. saudii* sp. nov., strain JCC^T^ (=CSUR P697 = DSM 27835) was grown anaerobically on 5% sheep blood-enriched Columbia agar (BioMerieux) at 37°C. *Bacteria* grown on three Petri dishes were harvested and resuspended in 4×100 μL of TE buffer. Then, 200 μL of this suspension was diluted in 1 ml TE buffer for lysis treatment that included a 30 minute incubation with 2.5 μg/μL lysozyme at 37°C, followed by an overnight incubation with 20 μg/μL proteinase K at 37°C. Extracted DNA was then purified using 3 successive phenol-chloroform extractions and ethanol precipitation at −20°C overnight. After centrifugation, the DNA was resuspended in 160 μL TE buffer. The yield and concentration was measured by the Quant-it Picogreen kit (Invitrogen) on the Genios-Tecan fluorometer.

### Genome sequencing and assembly

Genomic DNA of *C. saudii* was sequenced on a MiSeq instrument (Illumina Inc, San Diego, CA, USA) with 2 applications: paired end and mate pair. The paired end and the mate pair strategies were barcoded in order to be mixed respectively with 14 other genomic projects prepared with the Nextera XT DNA sample prep kit (Illumina) and 11 other projects with the Nextera Mate Pair sample prep kit (Illumina). The gDNA was quantified by a Qubit assay with the high sensitivity kit (Life technologies, Carlsbad, CA, USA) at 36.6 ng/μl and dilution was performed such that 1 ng of each genome was used to prepare the paired end library. The “tagmentation” step fragmented and tagged the DNA with a mean size of 1.5 kb. Then limited cycle PCR amplification (12 cycles) completed the tag adapters and introduced dual-index barcodes. After purification on AMPure XP beads (Beckman Coulter Inc, Fullerton, CA, USA), the libraries were then normalized on specific beads according to the Nextera XT protocol (Illumina). Normalized libraries were pooled into a single library for sequencing on the MiSeq. The pooled single strand library was loaded onto the reagent cartridge and then onto the instrument along with the flow cell. Automated cluster generation and paired end sequencing with dual index reads were performed in a single 39-hours run with a 2x250 bp read length. Total information of 5.3 Gb was obtained from a 574 K/mm^2^ cluster density with 95.4% (11,188,000) of the clusters passing quality control filters. Within this run, the index representation for *C. saudii* was determined to be 6.9%. The 710,425 reads were filtered according to the read qualities.

The mate pair library was prepared with 1 μg of genomic DNA using the Nextera mate pair Illumina guide. The genomic DNA sample was simultaneously fragmented and tagged with a mate pair junction adapter. The profile of the fragmentation was validated on an Agilent 2100 BioAnalyzer (Agilent Technologies Inc, Santa Clara, CA, USA) with a DNA 7500 labchip. The DNA fragments ranged in size from 1.4 kb up to 10 kb with a mean size of 5 kb. No size selection was performed and 600 ng of tagmented fragments were circularized. The circularized DNA was mechanically sheared to small fragments with a mean size of 625 bp on the Covaris device S2 in microtubes (Covaris, Woburn, MA, USA). The library profile was visualized on a High Sensitivity Bioanalyzer LabChip (Agilent Technologies Inc, Santa Clara, CA, USA). The libraries were normalized at 2 nM and pooled. After a denaturation step and dilution at 10 pM, the pool of libraries was loaded onto the reagent cartridge and then onto the instrument along with the flow cell. Automated cluster generation and sequencing run were performed in a single 42-hours run with a 2×250 bp read length.

Total information of 3.2 Gb was obtained from a 690 K/mm^2^ cluster density with 95.4% (13,264,000) of the clusters passing quality control filters. Within this run, the index representation for *C. saudii* was determined to be 8.2%. The 1,037,710 reads were filtered according to the read qualities.

### Genome annotation

Open Reading Frames (ORFs) were predicted using Prodigal [55] with default parameters. However, the predicted ORFs were excluded if they spanned a sequencing gap region. The predicted bacterial protein sequences were searched against the GenBank [56] and Clusters of Orthologous Groups (COG) databases using BLASTP. The tRNAs and rRNAs were predicted using the tRNAScanSE [57] and RNAmmer [58] tools, respectively. Lipoprotein signal peptides and numbers of transmembrane helices were predicted using SignalP [59] and TMHMM [60], respectively. Mobile genetic elements were predicted using PHAST [61] and RAST [62]. ORFans were identified if their BLASTP *E*-value was lower than 1e-03 for alignment length greater than 80 amino acids. If alignment lengths were smaller than 80 amino acids, we used an *E*-value of 1e-05. Such parameter thresholds have already been used in previous works to define ORFans. Artemis [63] and DNA Plotter [64] were used for data management and visualization of genomic features, respectively. Mauve alignment tool (version 2.3.1) was used for multiple genomic sequence alignment [65]. To estimate the Average Genome Identity of Orthologous Sequences (AGIOS) [7] at the genome level between *C. saudii* and another 9 members of the *Clostridium* genus (Table 3), orthologous proteins were detected using the Proteinortho [66] and we compared genomes two by two and determined the mean percentage of nucleotide sequence identity among orthologous ORFs using BLASTn.

**Table 3:** **Genomic comparison of *C. saudii *** **and 9 other members of *Clostridium* species**^**†**^

	***C. sma***	***C.bej***	***C. bot***	***C. car***	***C. cel***	***C. dak***	***C. dif***	***C. par***	***C. per***	***C. sen***
*C. sma*	**5,786**	1,479	1,181	1,034	1,779	1,100	1,037	1,554	1,351	1,137
*C. bej*	72.92	**4,911**	1,438	1,132	1,017	1,069	1,003	1,539	1,312	1,129
*C. bot*	71.34	73.00	**5,719**	1,533	1,275	1,101	1,099	1,046	1,337	1,210
*C. car*	71.11	71.66	73.13	**4,184**	1,426	1,334	1,182	1,162	1,294	1,252
*C. cel*	81.95	71.20	71.34	71.10	**4,066**	1,302	1,081	1,111	1,144	1,378
*C. dak*	70.13	70.38	71.06	71.46	74.04	**4,778**	1,149	1,119	1,076	1,137
*C. dif*	69.57	69.70	69.56	69.02	69.80	72.54	**3,553**	1,015	1,303	1,066
*C. par*	73.94	73.96	69.23	68.54	69.23	70.30	69.34	**3,244**	1,018	961
*C. per*	73.21	73.32	79.95	72.01	71.94	69.47	77.70	69.09	**4,485**	957
*C. sen*	71.94	72.07	71.53	71.10	73.11	72.16	70.40	71.58	69.58	**4,663**

^**†**^The numbers of orthologous protein shared between genomes (above diagonal), average percentage similarity of nucleotides corresponding to orthologous protein shared between genomes (below diagonal) and the numbers of proteins per genome (bold).

*C. sma = C. saudii*, *C.bej* = *C. beijerinckii*, *C. bot* = *C. botulinum*, *C. car* = *C. carboxidivorans*, *C. cel* = *C. celatum*, *C. dak* = *C. dakarense*, *C. dif* = *C. difficile*, *C. per* = *C. perfringens*, *C. par* = *C. paraputrificum*, *C. sen* = *C. senegalense*.

### Genome properties

**Figure 6:**
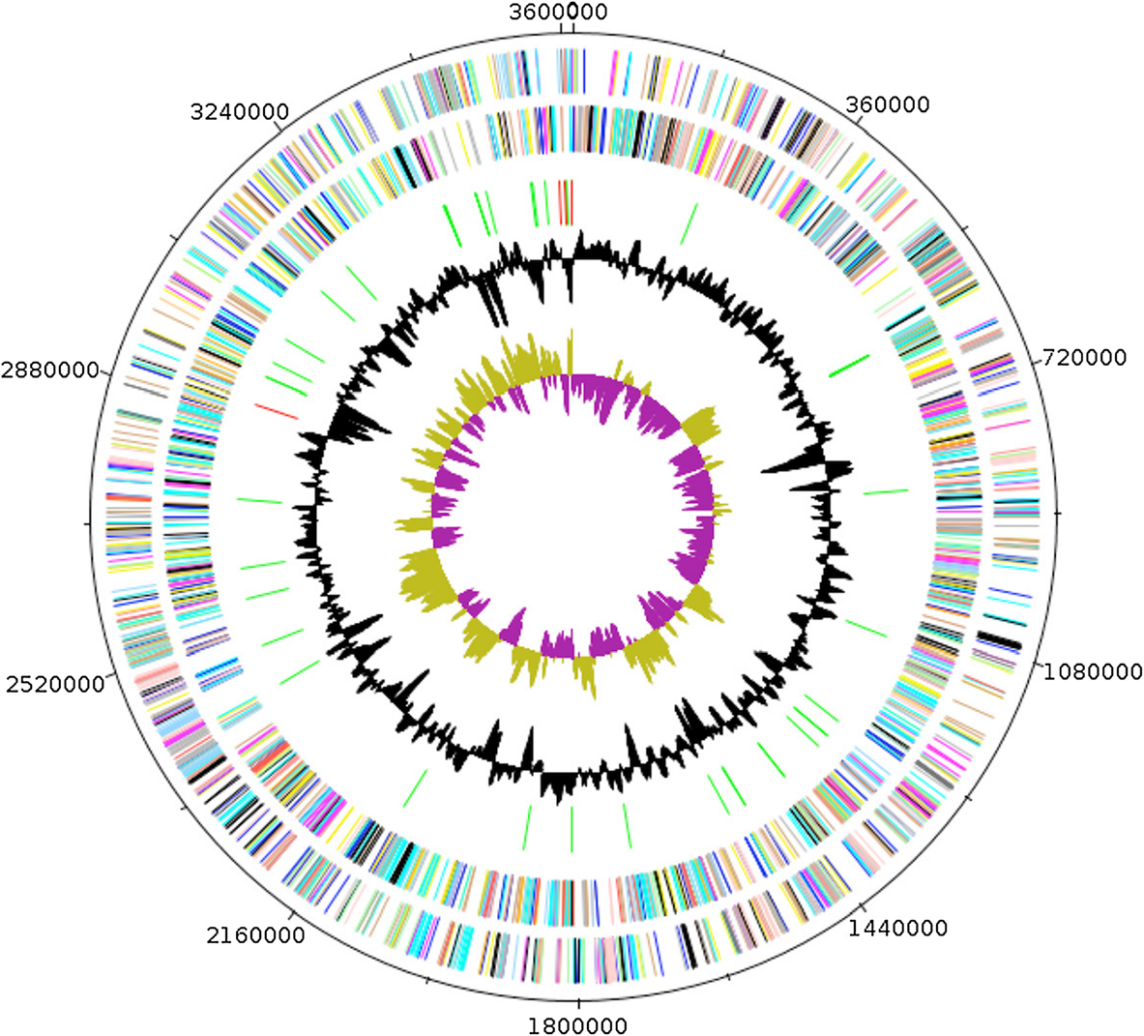
**Graphical circular map of the chromosome.** From outside to the center: Genes on the forward strand (colored by COG categories), genes on the reverse strand (colored by COG categories), RNA genes (tRNAs green, rRNAs red), GC content, and GC skew.

The genome is 3,653,762 bp long (one chromosome, no plasmid) with a GC content of 27.9% (Figure 6 and Table 4). Of the 3,509 predicted chromosomal genes, 3,452 were protein-coding genes and 57 were RNAs including 49 tRNAs and 8 rRNAs (5S = 6, 23S = 1, 16S = 1). A total of 2144 genes (61.10%) were assigned a putative function. One hundred and twenty eight genes were identified as ORFans (3.65%) and the remaining genes were annotated as hypothetical proteins. The properties and statistics of the genome are summarized in Tables 4 and 5. The distribution of genes into COGs functional categories is presented in Table 6.

**Table 4:** Nucleotide content and gene count levels of the genome

**Attribute**	**Value**	**% of total**^**a**^
Genome size (bp)	3,653,762	
DNA G + C content (bp)	1,019,399	27.9
DNA coding region (bp)	3,057,234	83.67
Total genes	3509	100
RNA genes	57	1.62
Protein-coding genes	3452	98.37
Genes with function prediction	2144	61.10
Genes assigned to COGs	2514	71.64
Genes with peptide signals	135	3.85
Genes with transmembrane helices	887	25.27

^a^The total is based on either the size of the genome in base pairs or the total number of protein coding genes in the annotated genome.

**Table 5:** Project information

**MIGS ID**	**Property**	**Term**
MIGS-31	Finishing quality	High-quality draft
MIGS-28	Libraries used	One paired-end 454 3-kb library
MIGS-29	Sequencing platforms	MiSeq Illumina
MIGS-31.2	Fold coverage	85.77×
MIGS-30	Assemblers	Newbler version 2.5.3
MIGS-32	Gene calling method	Prodigal
	GenBank ID	CBYM00000000
	GenBank Date of Release	February 12, 2014
MIGS-13	Project relevance	Study of the human gut microbiome

**Table 6:** Number of genes associated with the 25 general COG functional categories

**Code**	**Value**	**% age**^**a**^	**Description**
J	154	4.46	Translation
A	0	0	RNA processing and modification
K	296	8.57	Transcription
L	138	4	Replication, recombination and repair
B	1	0.03	Chromatin structure and dynamics
D	24	0.7	Cell cycle control, mitosis and meiosis
Y	0	0	Nuclear structure
V	73	2.11	Defense mechanisms
T	156	4.52	Signal transduction mechanisms
M	116	3.36	Cell wall/membrane biogenesis
N	62	1.8	Cell motility
Z	0	0	Cytoskeleton
W	0	0	Extracellular structures
U	48	1.4	Intracellular trafficking and secretion
O	66	1.91	Posttranslational modification, protein turnover, chaperones
C	153	4.43	Energy production and conversion
G	237	6.86	Carbohydrate transport and metabolism
E	328	9.5	Amino acid transport and metabolism
F	56	1.62	Nucleotide transport and metabolism
H	92	2.66	Coenzyme transport and metabolism
I	85	2.46	Lipid transport and metabolism
P	164	4.75	Inorganic ion transport and metabolism
Q	53	1.53	Secondary metabolites biosynthesis, transport and catabolism
R	346	10.02	General function prediction only
S	195	5.65	Function unknown
-	948	27.46	Not in COGs

^a^The total is based on the total number of protein coding genes in the annotated genome.

### Genome comparison of C. saudii with 9 other *Clostridium* genomes

**Figure 7:**
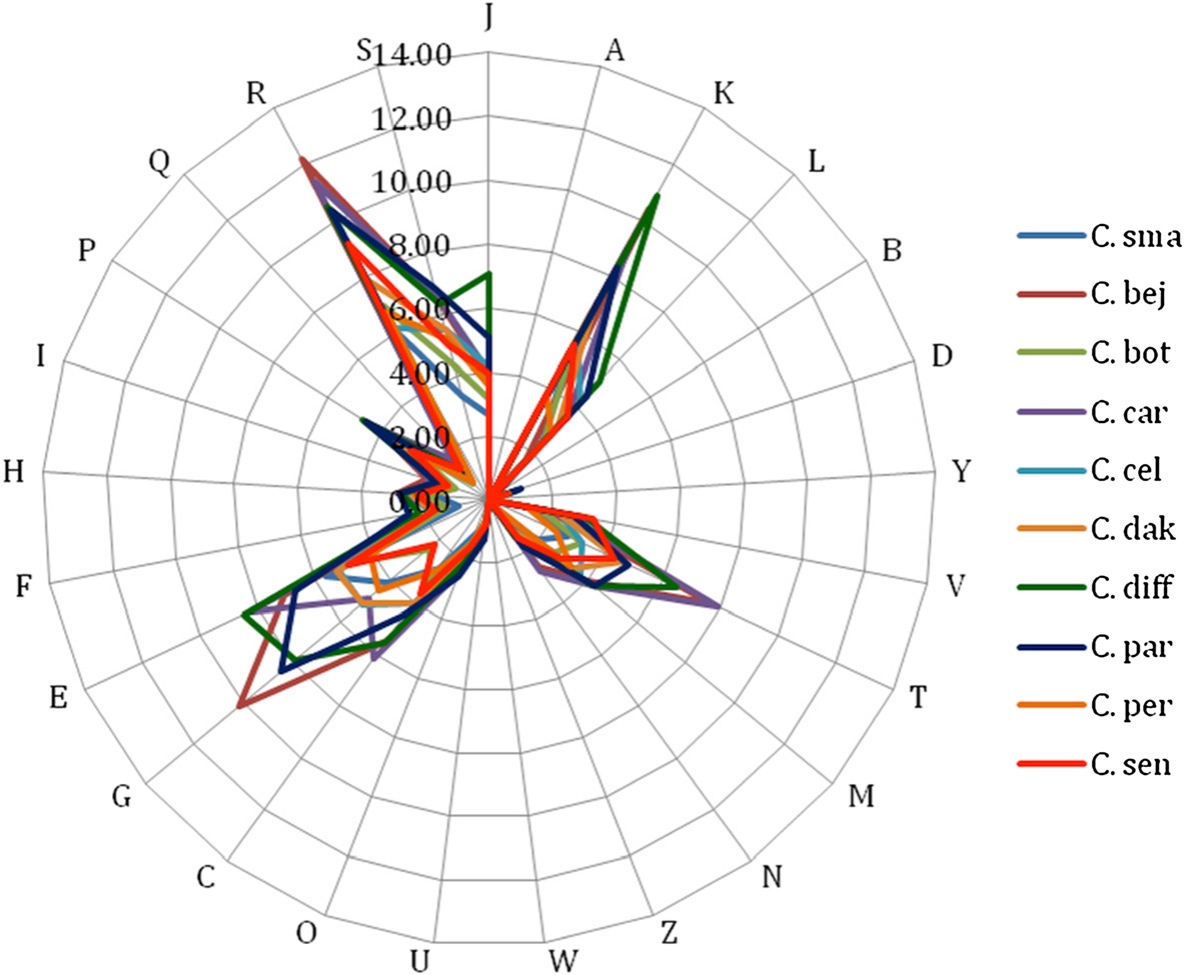
**Distribution of functional classes of predicted percentages of genes in the *C. saudii *JCC**^**T **^**and other 9 *clostridium *genomes according to the clusters of orthologous groups of proteins.***C.sma = C. saudii*, *C.bej* = *C. beijerinckii*, *C. bot* = *C. botulinum*, *C. car* = *C. carboxidivorans*, *C. cel* = *C. celatum*, *C. dak* = *C. dakarense*, *C. dif* = *C. difficile*, *C. per* = *C. perfringens*, *C. par* = *C. paraputrificum*, *C. sen* = *C. senegalense*.

We compared the genome of C. saudii strain JCC^T^ with those of *C. beijerinckii* strain NCIMB 8052, *C. botulinum* strain ATCC 3502, *C. carboxidivorans* strain P7, *C. celatum* strain DSM 1785, *C. dakarense* strain FF1, *C. difficile*s train B1, *C. perfringens* strain AGR 2156, *C. paraputrificum* strain ATCC 13124 and *C. senegalense* strain JC122 (Tables 6 and 7). The draft genome sequence of *C. saudii* strain JCC^T^ is smaller than those of *C. beijerinckii*, *C. botulinum*, *C. carboxidivorans*, *C. dakarense*, *C. difficile*, and *C. senegalense* (3.9, 4.41, 3.73, 4.46 and 3.89 Mb respectively), but larger than those of *C. celatum*, *C. paraputrificum* and *C. perfringens* (3.55, 3.56 and 3.26 Mb, respectively). The G + C content of *C. saudii* is lower than those of *C. beijerinckii*, *C. botulinum*, *C. carboxidivorans*, *C. dakarense*, *C. difficile*, *C. perfringens* and *C. paraputrificum* (29.0, 28.2, 29.7, 27.98, 28.4, 29.6 and 28.4%, respectively) but greater than those of *C. celatum* and *C. senegalense* (26.8 and 27.7 respectively). The gene content of *C. saudii* (3462) is smaller to those of *C. beijerinckii*, *C. botulinum*, *C. difficile*, *C. carboxidivorans*, *C. paraputrificum*, *C. dakarense* and *C. senegalense* (5020, 3590, 3934, 4174, 3568, 3843, and 3704 respectively) but larger that of *C. perfringens* and *C. celatum* (2876 and 3453 respectively). The distribution of genes into COG categories was almost similar in all the 10 compared genomes except the unique presence of cytoskeleton associated proteins in *C. difficile* (Figure 7).

**Table 7:** **Genomic comparison of *C. saudii *** **and 9 other members of *Clostridium* species**^**†**^

**Species**	**Strain**	**Genome accession number**	**Genome size (Mb)**	**G + C content**
*C. saudii*	JCC	In progress	3.65	27.9
*C. beijerinckii*	NCIMB 8052	NC_009617	6.0	29.0
*C. botulinum*	ATCC 3502	NC_009495	3.9	28.2
*C. carboxidivorans*	P7	NZ_ADEK00000000	4.41	29.7
*C. celatum*	DSM 1785	AMEZ01000000	3.55	27.7
*C. dakarense*	FF1	CBTZ010000000	3.73	27.98
*C. difficile*	B1	NC_017179	4.46	28.4
*C. paraputrificum*	AGR2156	AUJC01000000	3.56	29.6
*C. perfringens*	ATCC 13124	NC_008261	3.26	28.4
*C. senegalense*	JC122	CAEV01000001	3.89	26.8

^†^Species name, Strain, GenBank accession number, Genome size and GC content of genomes compared.

In addition, *C. saudii* shared 1479, 1181, 1034, 1779, 1100, 1037, 1554, 1351, and 1137 orthologous genes with *C. beijerinckii*, *C. botulinum*, *C. carboxidivorans*, *C. celatum*, *C. dakarense*, *C. difficile*, *C. perfringens*, *C. paraputrificum* and *C. senegalense*, respectively. Among compared genomes AGIOS values ranged from 68.54 between *C. carboxidivorans* and *C. paraputrificum* to 79.95% between *C. botulinum* and *C. perfringens*. When *C. saudii* was compared to other species, AGIOS values ranged from 69.57 with *C. difficile* to 81.95% with *C. celatum* (Table 7).

## Conclusion

On the basis of phenotypic, phylogenetic and genomic analyses, we formally propose the creation of *Clostridium saudii* sp. nov. that contains the strain JCC^T^. This bacterial strain was isolated in Marseille, France.

### Description of *Clostridium saudii* sp. nov

*Clostridium saudii* (sa.u'di.i N.L. gen. n. saudii, of Saudi Arabia, for the country where the strain originates). Isolated from an obese Saudi patient sample. Transparent colonies were 0.2 to 0.3 mm in diameter on blood-enriched agar. *C. saudii* is a Gram-positive, obligate anaerobic, endospore-forming bacterium with a mean diameter of 1 μm. Optimal growth was observed at 37°C. *C. saudii* is catalase and oxidase negative. Alpha-galactosidase, β-galactosidase, β-galactosidase-6-phosphatase, α-glucosidase, β-glucosidase, α-arabinosidase, N-acetyl-β-glucosaminidase, alkaline phosphatase, arginine arylamidase, pyroglutamic acid arylamidase, tyrosine arylamidase, alanine arylamidase, glycine arylamidase and histidine arylamidase were positive. Urease, arginine dihydrolase, β-glucuronidase, fermentation of mannose and raffinose, glutamic acid decarboxylase, α-fucosidase, nitrate reduction, indole production, proline arylamidase, leucyl glycine arylamidase, phenylalanine arylamidase, leucine arylamidase, glutamyl glutamic acid arylamidase and serine arylamidase were negative. Asaccharolytic. *C. saudii* is susceptible to imipenem, trimethoprim-sulfamethoxazole, metronidazole, doxycycline, rifampicin, vancomycin and amoxicillin-clavulanate and resistant to amoxicillin, ciprofloxacine, erythromycin and gentamicin.

The G + C content of the genome is 28%. The 16S rRNA and genome sequences are deposited in GenBank under accession numbers HG726039 and CBYM00000000, respectively. The type strain is JCC^T^ (=CSUR P697 = DSM 27835).

## Competing interests

The authors declare that they have no competing interests.

## Authors’ contributions

EA: wrote the manuscript and analysed the data; FB: analysed the data; DhR: analysed the genome; EIA: organized the study in Saudi Arabia; AJF: collected samples in Saudi Arabia; SA: collected samples in Saudi Arabia; JCL: cultured the samples and analyzed microbiological data; CR: performed the sequencing analysis; AC: analyzed sequences; MH: collected data in Saudi Arabia; PEF: organized the study and wrote the manuscript; DR: organized the study.
